# Developing an Evidence-Based Fall Prevention Curriculum for Community Health Workers

**DOI:** 10.3389/fpubh.2014.00209

**Published:** 2015-04-27

**Authors:** Julie A. St. John, Tiffany E. Shubert, Matthew Lee Smith, Cherie A. Rosemond, Doris A. Howell, Christopher E. Beaudoin, Marcia G. Ory

**Affiliations:** ^1^Department of Public Health, Graduate School of Biomedical Sciences, Texas Tech University Health Sciences Center, Abilene, TX, USA; ^2^Division of Geriatrics, School of Medicine, University of North Carolina at Chapel Hill, Chapel Hill, NC, USA; ^3^College of Public Health, The University of Georgia, Athens, GA, USA; ^4^Center for Health Promotion and Disease Prevention, University of North Carolina at Chapel Hill, Chapel Hill, NC, USA; ^5^Program on Healthy Aging, Texas A&M Health Science Center School of Public Health, College Station, TX, USA; ^6^Department of Communication, Texas A&M University, College Station, TX, USA; ^7^Department of Health Promotion and Community Health Sciences, Texas A&M Health Science Center School of Public Health, College Station, TX, USA

**Keywords:** community health workers, promotores, curriculum development, training, fall prevention, older adults

## Abstract

This perspective paper describes processes in the development of an evidence-based fall prevention curriculum for community health workers/promotores (CHW/P) that highlights the development of the curriculum and addresses: (1) the need and rationale for involving CHW/P in fall prevention; (2) involvement of CHW/P and content experts in the curriculum development; (3) best practices utilized in the curriculum development and training implementation; and (4) next steps for dissemination and utilization of the CHW/P fall prevention curriculum. The project team of CHW/P and content experts developed, pilot tested, and revised bilingual in-person training modules about fall prevention among older adults. The curriculum incorporated the following major themes: (1) fall risk factors and strategies to reduce/prevent falls; (2) communication strategies to reduce risk of falling and strategies for developing fall prevention plans; and (3) health behavior change theories utilized to prevent and reduce falls. Three separate fall prevention modules were developed for CHW/P and CHW/P Instructors to be used during in-person trainings. Module development incorporated a five-step process: (1) conduct informal focus groups with CHW/P to inform content development; (2) develop three in-person modules in English and Spanish with input from content experts; (3) pilot-test the modules with CHW/P; (4) refine and finalize modules based on pilot-test feedback; and (5) submit modules for approval of continuing education units. This project contributes to the existing evidence-based literature by examining the role of CHW/P in fall prevention among older adults. By including evidence-based communication strategies such as message tailoring, the curriculum design allows CHW/P to personalize the information for individuals, which can result in an effective dissemination of a curriculum that is evidence-based and culturally appropriate.

## Introduction

“I’ve fallen and I can’t get up,” was a phrase made popular by LifeCall in 1989. This commercial was a dramatized version of an older adult’s fall. However, this situation is the reality for numerous older adults in the United States. Falls are a threat to the lives, independence, and health of adults – especially those aged 65 and older. Every 18 s an older adult visits an emergency department as a result a fall, and every 35 min an older adult dies due to injuries from such a fall (1–7).

Other significant consequences are associated with falls. For example, one in three adults aged 65 and older fall each year, costing the U.S. healthcare system more than $30 billion dollars annually (1). This problem is even more significant due to the rapidly expanding aging population (8). In light of the rate of falls among older adults, physical and fiscal costs, severity of falls, and population growth among adults ages 65 and older, researchers are investigating how to effectively prevent and reduce falls among older adults.

Research has demonstrated that a large proportion of falls among community-dwelling older adults are preventable (9, 10). Numerous documented strategies address fall prevention among older adults – community programing, primary care practice guidelines, and integration of physical therapists into models of care (11). Despite the growth of evidence-based fall prevention programs and the emergence of state-wide fall prevention policy efforts, there continues to be a gap in community adoption of fall prevention interventions among underserved, rural, minority, and low-income populations (12). Literature is lacking regarding: (1) the reasons why fall prevention policies and programs are or are not adopted and spread in community settings; (2) the most efficient practices for creating a trained workforce for delivering interventions; (3) the best strategies for reaching out to underserved populations in terms of recruitment, geographic, and needs-based challenges; and (4) how to connect community and clinical care settings (12). A lack of infrastructure for disseminating and implementing interventions to community-based programs has contributed significantly to this gap.

The Policies, Programs, and Partners for Fall Prevention (PPPFP) study incorporated multi-level intervention strategies to develop several dissemination approaches (13). This paper focuses on a training Community Health Workers/Promotores (CHW/P) to deliver fall prevention messages to older adults. CHW/P are described as frontline public health workers, serving as liaisons between health and social services and the community. They facilitate access and improve the quality and cultural competence of service delivery by utilizing a wide array of skill sets (14–17). CHW/P are trusted members of the target community, work for pay or as volunteers, and typically share ethnicity, language, socioeconomic status, and life experiences with the community members served. As such, CHW/P can communicate with other members of the healthcare system to ensure that community members’ care is sensitive to cultural and community issues. Research has demonstrated the effectiveness of CHW/P among targeted Hispanic populations in achieving positive health outcomes through health education, case management, service coordination, and referrals (18–31). Specifically, CHW/P are effective due to their cultural similarity and understanding of the population they serve, as well as the subsequent trust clients have in them. These CHW/P characteristics are largely due to their residing in the same communities. Further, studies have demonstrated the effectiveness of CHW/P in providing social support to help Hispanics adopt behavior change (24–30).

One of the greatest challenges in effective fall prevention is ensuring the population at risk actually receives recommended interventions. CHW/P can serve as liaisons for older adults at risk for falling, making sure they are referred to accessible services and helping make sure interventions at the community or healthcare level are supported to maximize program adherence. The literature supports CHW/P as a conduit to increase fall prevention awareness, but there is a gap in fall prevention training for CHW/P. This perspective paper will describe the development and implementation of the fall prevention curriculum for CHW/P with a focus on: (1) making the case for developing evidence-based CHW fall prevention training; and (2) explaining programmatic activity, including informal focus groups, module development, pilot testing, curriculum refinement, continuing education units (CEUs) approval, and next steps in dissemination.

## Making the Case for Developing an Evidence-Based CHW Fall Prevention Training

A preparatory national scan of CHW/P curriculum, trainings, and resources about fall prevention among older adults was conducted in October–December 2011, revealing a lack of a comprehensive fall prevention curricula specifically designed for CHW/P. The scan included searches conducted on web-based search engines and phone calls and emails to CHW/P organizations, networks, associations, state CHW program offices, employers, and academic institutions. No curricula on fall prevention for older adults specifically for CHW/P were located. A wider search revealed an evidence-based curriculum to train nurse assistants in fall prevention in home health settings, which has been available since 2007. Based on the best practice strategies discussed in detail in the following section, along with fall prevention strategies, we developed a series of CHW/P curriculum entitled, “How can CHW/Promotores help older adults stay safe from falls and related injuries?” The curriculum was developed by the study team from October 2012 to May 2013 and piloted in McAllen, Texas, on June 4–5, 2013, in English and Spanish with 49 CHW/P. Revisions were made from July 2013 to December 2013, and the revised curriculum was deployed nationally in face to face and virtual formats in April 2014. This project was approved by the Institutional Review Board at Texas A&M University.

### Employment of best practice approaches

To effectively develop and train CHW/P, we employed three best practices: (1) utilization of CHW/P to deliver health education messages; (2) adult learning theory; and (3) tailored messaging. Studies have demonstrated CHW/P are effective in delivering health education to community residents due to shared ethnicity, language, socioeconomic status, and life experiences with the community members they serve (18–31). The project team included CHW/P throughout the development of processes to train and engage CHW/P to deliver fall prevention health education messages. Specifically, CHW/P identified the needs of their communities, reviewed the training materials, and identified gaps in information and service.

Second, an adult learner-centered training approach that considers characteristics of the target audience was utilized. CHW/P are typically between the ages of 20 and 65, have lower educational attainment with reading and math skills ranging between 4th and 8th grade levels, and are non-native English speakers. Adult learner-centered educational strategies engage learners in problem-based learning and teaching. Rather than a “lecture,” learner-centered approaches engage learners in hands-on, interactive activities based upon discussion and skill-building exercises (32–35).

Third, tailored messaging was incorporated into the CHW/P fall prevention curriculum. Message tailoring deploys information and change strategies to reach one specific person based on the individual characteristics (36, 37). Tailoring differs from targeting of general audiences and segmenting of subgroups by customizing (or personalizing) educational approaches and messages to the individual. CHW/P were trained to employ tailoring to effectively educate clients. Instead of providing general education to their overall audience – or more refined education to certain subgroups within that overall audience – CHW/P made assessments about and delivered education based on the characteristics of individuals in their constituency, including culture, language, health literacy, education, gender, age, and pertinent experiences, beliefs, and attitudes. Tailoring-based approaches acknowledge how individuals differentially use, learn, and benefit from varied educational and messaging approaches. The purpose of developing training with integrated tailored messaging taught via adult learning strategies was to support CHW/P to utilize their strengths to ensure optimization of fall prevention interventions.

## Explaining Programmatic Activity

### Informal focus groups to identify need

**Figure 1:**
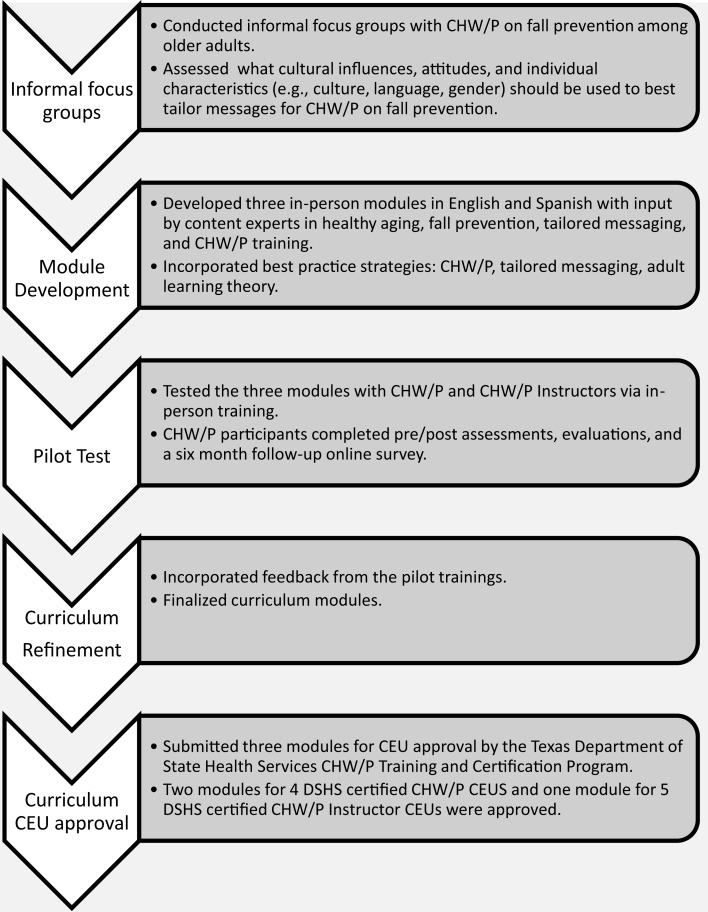
**Process of CHW/P fall prevention training module development**.

The lead partner was a Texas Department of State Health Services (DSHS) Certified CHW/P Training Center. The curriculum incorporated the eight competencies recognized by the Texas DSHS CHW/P Training and Certification Program: (1) communication; (2) teaching; (3) advocacy; (4) interpersonal skills; (5) service coordination; (6) capacity building; (7) organization; and (8) knowledge-based skills. Texas certifies CHW/P and requires a minimum of 20 CEUs (i.e., 10 DSHS-certified and 10 non-certified CEUs) every 2 years for recertification. To maximize adoption, the project team developed the curriculum in English and Spanish and met DSHS requirements for CEUs for CHW/P in Texas. Figure 1 depicts the five-step training development module process, which is discussed in detail in the ensuing paragraphs.

Prior to material development, the project team conducted informal conversations with CHW/P about fall prevention (Step 1). These conversations identified potential gaps in knowledge to inform curriculum development and identify cultural influences and attitudes critical for message tailoring. This procedure ensured the training modules integrated input from the CHW/P and created a framework to integrate characteristics unique to CHW/P (e.g., culture, language, and gender) to best tailor messages.

### Module development

The feedback from the informal focus groups and input by content experts in healthy aging, fall prevention, health messaging, and CHW/P training contributed to developing and refining tailoring-based training modules in English and Spanish. The curriculum was developed for any CHW/P instructor (whether DSHS-certified or not) and those with varying knowledge about fall prevention (whether a novice or expert). The intent was for any CHW/P to use the curriculum and training guide to train other CHW/P about fall prevention among older adults. Specific objectives included:

- Explain that falls are not a normal part of aging and the majority of falls are preventable.
- Articulate why preventing and reducing falls and related injuries among older people is especially important.
- Increase awareness about risk factors for falling inside and outside the home.
- Develop and augment observation, reporting, and communication skills to improve communication with older adults and their families around fall prevention.
- Describe ways to help prevent or manage falls in older adults.
- Develop fall prevention plans.
- Explain and discuss different approaches to changing health behaviors.
- Apply behavior change strategies to fall prevention and reduction.
- Teach health behavior change strategies to CHW/P and older adults.

As shown in Table 1, the three in-person training modules addressed the following topics: (1) fall risk factors and strategies to reduce/prevent falls (4 h in length); (2) communication strategies to reduce risk of falling and develop fall prevention plans (4 h in length); and (3) using health behavior change theories to prevent and reduce falls (5 h in length). The first two modules were designed to be completed independently but were linked in theme and content. The third module focuses on equipping CHW/P Instructors to apply health behavior theory to fall prevention and reinforces fall risk factors and strategies to prevent falls and related injuries.

**Table 1:** **Contents of CHW/P fall prevention training modules**.

Fall prevention: curriculum for community health workers/promotores			
How can CHW/promotores help older adults stay safe from falls and related injuries?			
Session title	Content outline	Target audience	Session length
Session 1: ways to prevent falls and related injuries in older adults	1. Statistics on falls among older adults	CHW/P	4 h/4 CEUs
	a. Why talk about fall prevention?	
	b. Goals of the session	
	c. The facts on falls	
	1) U.S. falls statistics	
	2) Local falls statistics	
	2. Common risk factors for falling	
	a. Individual risk factors	
	1) Physical mobility	
	2) Medications	
	3) Transitioning home from hospital	
	4) Fear of falling	
	5) Traumatic brain injury (TBI)	
	6) Cognitive impairment	
	a. Environmental risk factors	
	1) Home safety	
	2) Safety factors outside the home	
	3. How to identify and address risk factors	
	a. Assessments	
	1) Home fall prevention assessment for older adults	
	2) Check your risk for falling – self assessment	
	b. Communication strategies	
	Closed and open-ended questions	
	4. How to prevent and reduce falls	
	a. Prevention tips	
	1) Regular exercise program	
	2) Have healthcare provider review medications	
	3) Have vision checked	
	4) Make home safer	
	b. Putting information into action	
	1) Case studies & role play	
	2) Identification of resources	
Session 2: learning skills to reduce falls and related injuries	1. Risk factors for falls	CHW/P	4 h/4 CEUs
	a. Individual risk factors	
	1) Physical mobility	
	2) Medications	
	3) Transitioning home from hospital	
	4) Fear of falling	
	5) Traumatic brain injury (TBI)	
	6) Cognitive impairment	
	b. Environmental risk factors	
	1) Home safety	
	2) Safety factors outside the home	
	2. Enhance communication skills to tailor messages and ask open-ended questions	
	a. Strategies to communicate effectively about falls prevention	
	1) Closed and open-ended questions	
	2) Observe, record, and report	
	3) Communicating with older adults	
	a) What to do if an older adult falls	
	b) How to get up from a fall	
	4) Communicating with care givers	
	5) Communicating with health professionals	
	b. Tailored messaging	
	1) Assessment	
	a) Stage of behavioral change of the individual	
	b) Personal characteristics of the individual	
	2) Message creation and delivery	
	a) Analyze falls prevention strategies	
	b) Identify characteristics	
	c) Develop relevant messages	
	3. Apply communication skills to case scenarios and identify risk factors to reduce risk of falling	
	a. Case studies	
	b. Role play	
	4. Develop and implement a fall prevention plan	
	a. Role play	
	b. Interview an older adult	
	5. Identify resources for fall prevention	
	a. Group and individual activities	
Session 3: helping older adults change their health behaviors to prevent falls and related injuries: health behavior change theories	1. Theories of health behavior change	CHW/P instructors	5 h/5 CEUs
	a. Why talk about behavior change	
	b. Health belief model	
	1) Constructs	
	a) Perceived susceptibility	
	b) Perceived severity	
	c) Perceived benefits	
	d) Perceived barriers	
	e) Perceived self-efficacy	
	f) Cues to action	
	2) Scenarios	
	c. Trans-theoretical model	
	1) Stages of change	
	a) Pre-contemplation	
	b) Contemplation	
	c) Preparation	
	d) Action	
	e) Maintenance	
	2) Processes of change	
	3) Scenarios	
	d. Theory of reasoned action/theory of planned behavior	
	1) Constructs	
	a) Attitude	
	b) Norm	
	c) Intention	
	d) Perceived behavioral control	
	2) Scenarios	
	2. Fall prevention risk factors	
	a. Individual risk factors	
	1) Physical mobility	
	2) Medications	
	3) Transitioning home from hospital	
	4) Fear of falling	
	5) Traumatic brain injury (TBI)	
	6) Cognitive impairment	
	b. Environmental risk factors	
	1) Home safety	
	2) Safety factors outside the home	
	3. Strategies for managing falls	
	a. Regular exercise program	
	b. Have healthcare provider review meds	
	c. Have vision checked	
	d. Make home safer	
	4. Tailored communication	
	a. Communication approaches	
	1) General messaging	
	2) Targeted messaging	
	3) Segmented messaging	
	4) Tailored messaging	
	b. Reasons to tailor messages	
	c. Research on tailoring	
	d. Steps in tailoring	
	1) Analyze a health problem	
	2) Identify pertinent characteristics	
	3) Develop pertinent messaging	
	e. Key fall prevention messages	
	5. Application of behavior change concepts to fall prevention and reduction	
	a. Review game	
	b. Case studies	
	6. Skills to work with older adults/CHW/P to implement behavioral changes to prevent falls	
	a. Interviews with older adults	
	b. Practice assessments	

Course materials included: an introduction for trainers, facilitator’s guides, participant handouts, case studies, pre/post-assessments, and evaluations. Pre/post-assessments measured knowledge and confidence related to fall prevention and communication strategies. The evaluations gathered participant demographic information and satisfaction with the training. The curriculum incorporated teaching methods focusing on increasing self-awareness and skill building through practical application, including case scenarios, role play, group work, and interactive presentations. Refer to Table 1 for detailed content.

### Pilot testing

After developing the modules, each module was pilot tested with a group of CHW/P and CHW/P Instructors (i.e., 44 participants for Module 1; 41 for Module 2; and 18 for Module 3). CHW/P and CHW/P Instructors were recruited via emails and word-of-mouth. Two DSHS-certified, experienced, bilingual, and bicultural CHW/P Instructors conducted the in-person trainings in English and Spanish – with all materials provided in both languages. The pilot included evaluation and assessment onsite after completion of each training module and an online survey deployed 6 months after training to assess knowledge and implementation in their roles as CHW/P.

### Curriculum refinement

The team refined the curriculum based on feedback from the pilot test. Specific feedback and revisions to the final curriculum included:

(1) Standardized wording for CHW/P and fall prevention terminology.
(2) Refined case studies.
(3) Added detailed physical activity exercises.
(4) Added a handout on local, state, and national resources; a glossary of terms; and a fall-related Frequently Asked Questions (FAQ) handout.

### Continuing education units approval

The final step of the curriculum development process included submitting the three modules in English and Spanish for CEU approval by the Texas DSHS CHW/P Training and Certification Program. Each of the three models was approved. Information regarding the CEU approval process can be found at http://www.dshs.state.tx.us/mch/chw.shtm; information regarding the CHW/P fall prevention modules may be found at http://nchwtc.tamhsc.edu/.

### Next steps in dissemination

The curriculum was converted into an online format for broader dissemination (http://nchwtc.tamhsc.edu/fall-prevention-curriculum). The online formats include two courses:

1. *CHW/P Course*: Preparing CHWs/Promotores to prevent and reduce falls among older.
2. *CHW/P Instructor Course*: Helping older adults change their health behaviors to prevent falls and related injuries: health behavior change theories.

For the online format, CHW/P complete pre/post-assessments, an evaluation, and a 6-month follow-up survey. The goal of this approach is to create a feasible and sustainable training method that minimizes resources while maximizing dissemination – particularly in rural and remote communities that have CHW/P but do not have local CHW/P training programs. This strategy is designed to support implementation sustainability because CHW/P who received training from the fall prevention modules can continue to revisit these modules at no incurred cost in their future health outreach, education, and promotion strategies.

## Discussion

This perspective paper describes the development of a fall prevention curriculum for CHW/P. Given the access of CHW/P to at-risk older adults and their effectiveness to educate and promote behavior change, CHW/P are logical partners in promoting fall prevention strategies. However, to date, little has been attempted to engage CHW/P in fall prevention interventions, despite the scope of the problem. More specifically, there has not been another evidence-based curriculum on fall prevention among older adults specifically designed for CHW/P.

Numerous studies have highlighted the utilization of CHW/P and their effectiveness in helping their target populations achieve positive health outcomes through health education, promotion, and outreach (17–31). CHW/P are effective in these roles due to their cultural similarity and understanding of the population they serve and the subsequent trust that residents have in them (17–31). Specifically, CHW/P-led educational interventions have led to increased participant self-efficacy, knowledge, and adoption of preventive behaviors (38–43). Further, studies have demonstrated the effectiveness of CHW/P in providing social support to help participants adopt behavior change (38–40). One intent of this project was to build on the literature that has demonstrated the effectiveness of CHW/P in educational interventions to improve knowledge and adoption of behavior changes by the target population to include fall prevention education and promotion for older adults, which previously was a gap in the literature, given the significant burden and cost of falls by older adults in the U.S.

An innovative aspect of the study was to actively engage CHW/P in all stages of the curriculum development, which included pilot testing, refining, and implementation. The curricula utilized best practice strategies of CHW/P, adult learning theory, and tailored messaging, as well as evidence-based fall prevention strategies. The structured engagement of CHW/P during the development process had many benefits. First, the included messages, content, and format of the curricula were relevant, acceptable, and comprehendible for the CHW/P and CHW/P Instructors. Second, the messages, content, and format were appropriate for older adults who would be reached by the fall prevention activities. Third, by vetting the modules among the intended community, the goal of widespread adoption, dissemination, and sustainability was more realistic and obtainable. A potential limitation of the CHW/P curriculum on fall prevention among older adults is that the impact of the curriculum on reducing falls and injuries caused by falls by older adults could depend on the actual implementation of the curriculum – relying on the capacity of CHW/P programs, trainers, and employers – rather than on the actual curriculum. The project team anticipated this limitation and attempted to address this potential limitation through detailed instructions, tools, and resources within the curriculum and through providing additional technical assistance upon request to CHW/P, CHW/P instructors or trainers, and CHW/P employers on how to utilize and implement the curriculum with CHW/P and older adults.

The future directions of this project as a translated curriculum in an online format has great potential to broaden its reach and further disseminate the modules – particularly in rural areas where CHW/P training programs are scarce and communities have a greater population of older adults. Key will be to assess the ease of use, esthetics, and platform selected for online content to ensure CHW/P can successfully access and complete the online modules and revisit resources and training materials. Careful and strategic selection of partners for promoting and supporting this internet-based curricula can substantially impact uptake and utilization. The project team members will rely on existing partners and collaborators to broadly disseminate the self-paced, online training modules available for CHW/P and CHW/P Instructors in English or Spanish.

## Conclusion

This perspective paper describes the process of evaluating the CHW/P training curriculums, identifying a gap in the curriculum, and then the process of developing a CHW/P curriculum that specifically targets management of fall prevention. The goal of the curriculum is to equip and mobilize CHW/P so they can play a key role in joining other public health professionals in the fight to prevent and reduce injuries among older adults due to falling. In other areas, CHW/Ps have proven effective in helping individuals adopt preventive behaviors through educational interventions (38–43). The curriculum development was the first step in determining if a similar model could be used for fall prevention. The process we undertook validated that CHW/Ps had a need for this type of curriculum, were interested in the curriculum, and could improve their knowledge by participating in the curriculum. Engaging CHW/P remains especially vital in that CHW/P have a unique opportunity to reach out to older adults who may otherwise be neglected and have an increased risk for falling.

## Conflict of Interest Statement

The authors declare that the research was conducted in the absence of any commercial or financial relationships that could be construed as a potential conflict of interest.

This paper is included in the Research Topic, “Evidence-Based Programming for Older Adults.” This Research Topic received partial funding from multiple government and private organizations/agencies; however, the views, findings, and conclusions in these articles are those of the authors and do not necessarily represent the official position of these organizations/agencies. All papers published in the Research Topic received peer review from members of the Frontiers in Public Health (Public Health Education and Promotion section) panel of Review Editors. Because this Research Topic represents work closely associated with a nationwide evidence-based movement in the US, many of the authors and/or Review Editors may have worked together previously in some fashion. Review Editors were purposively selected based on their expertise with evaluation and/or evidence-based programming for older adults. Review Editors were independent of named authors on any given article published in this volume.
